# Digital Interventions to Improve Health Literacy Among Parents of Children Aged 0 to 12 Years With a Health Condition: Systematic Review

**DOI:** 10.2196/31665

**Published:** 2021-12-22

**Authors:** Evalotte Mörelius, Suzanne Robinson, Diana Arabiat, Lisa Whitehead

**Affiliations:** 1 School of Nursing and Midwifery Edith Cowan University Joondalup, WA Australia; 2 Department of Health, Medicine and Caring Sciences Linköping University Linköping Sweden; 3 The Centre for Evidence Informed Nursing, Midwifery and Healthcare Practice Joondalup, WA Australia; 4 Maternal and Child Nursing Department Faculty of Nursing The University of Jordan Amman Jordan; 5 Australian Research Council Centre of Excellence for the Digital Child Joondalup, WA Australia

**Keywords:** child, child health services, digital technology, health literacy, infant, internet-based intervention, parents, patient compliance, pediatric hospitals

## Abstract

**Background:**

Parental health literacy is associated with child health outcomes. Parents are increasingly turning to the internet to obtain health information. In response, health care providers are using digital interventions to communicate information to assist parents in managing their child’s health conditions. Despite the emergence of interventions to improve parental health literacy, to date, no systematic evaluation of the effectiveness of the interventions has been undertaken.

**Objective:**

The aim of this review is to examine the effect of digital health interventions on health literacy among parents of children aged 0-12 years with a health condition. This includes evaluating parents’ engagement (use and satisfaction) with digital health interventions, the effect of these interventions on parental health knowledge and health behavior, and the subsequent impact on child health outcomes.

**Methods:**

This systematic review was registered a priori on PROSPERO (International Prospective Register of Systematic Reviews) and developed according to the Joanna Briggs Institute methodology for systematic reviews. The databases CINAHL, MEDLINE, and PsycINFO were searched for relevant literature published between January 2010 and April 2021. Studies were included if they were written in English. A total of 2 authors independently assessed the search results and performed a critical appraisal of the studies.

**Results:**

Following the review of 1351 abstracts, 31 (2.29%) studies were selected for full-text review. Of the 31 studies, 6 (19%) studies met the inclusion criteria. Of the 6 studies, 1 (17%) was excluded following the critical appraisal, and the 5 (83%) remaining studies were quantitative in design and included digital health interventions using web-based portals to improve parents’ health knowledge and health behavior. Owing to heterogeneity in the reported outcomes, meta-analysis was not possible, and the findings were presented in narrative form. Of the 5 studies, satisfaction was measured in 3 (60%) studies, and all the studies reported high satisfaction with the digital intervention. All the studies reported improvement in parental health literacy at postintervention as either increase in disease-specific knowledge or changes in health behavior. Of the 5 studies, only 1 (20%) study included child health outcomes, and this study reported significant improvements related to increased parental health knowledge.

**Conclusions:**

In response to a pandemic such as COVID-19, there is an increased need for evidence-based digital health interventions for families of children living with health conditions. This review has shown the potential of digital health interventions to improve health knowledge and behavior among parents of young children with a health condition. However, few digital health interventions have been developed and evaluated for this population. Future studies with robust research designs are needed and should include the potential benefits of increased parent health literacy for the child.

**Trial Registration:**

PROSPERO International Prospective Register of Systematic Reviews CRD42020192386; https://www.crd.york.ac.uk/prospero/display_record.php?RecordID=192386

## Introduction

### Background

Parents of young children are responsible for the health and well-being of their children and are the advocates and primary caregivers of their children [1]. Parents are expected to interact with health services in the delivery of health care [2] and learn about their child’s health condition and potential interventions and procedures involved in treatment [3,4]. To interact effectively with health services, parents of children with health conditions need a level of health literacy. Health literacy increases the parents’ capacity to take responsibility and be involved in making decisions related to their child’s health [2].

Health literacy is commonly described as knowledge, motivation, and competence to assess, understand, appraise, and apply health information. Health literacy enables people to make decisions on health care, disease prevention, and health promotion throughout their life course [5]. A parent’s level of health literacy influences 3 aspects of health behavior: access and use of health services, patient-provider interactions, and self-management [5].

Low parental health literacy has been linked to poor health knowledge and child health status [6-9]. Limited health literacy has been associated with various concerns, including delayed diagnoses, misunderstanding of medication labels [10,11], poor adherence to treatment regimens, and increased use of emergency care [12]. Limited parental health literacy may also result in poor child health outcomes, including depressive symptoms, persistent asthma [8,13-16], and less than optimal glycemic control in children with diabetes [17].

Parents’ need for health information can remain unmet [18]. Moreover, parents of children with health conditions are often affected by stress [19] and poor sleep, which may hinder their ability to receive and process new information and learn about their child’s condition to provide them with optimal care [20,21]. Information about a child’s health condition needs to be delivered at a time that is appropriate for the family and offered as many times as needed, which can be challenging for both the parents and the health service [22,23]. A way to enhance the delivery of information is through digital technology (eg, mobile phones and tablets), as this can be accessed at a time, place, and pace that best suits the parent. Thus, digital health interventions (eg, information videos, web-based platforms, and mobile apps) can be used in the home settings where most care is provided [3]. Internet and mobile phone use are high among parents worldwide, with most parents using the internet and mobile phones to access information multiple times a day [24,25]. Parents are heavy users of web-based child health-related information [25]. Between 70% and 80% of parents have searched on the web for health information, with most parents seeking parenting advice, health information, or social support [26].

Health care providers increasingly use digital technologies to communicate information to address health needs and deliver health care interventions [27]. Despite minimal evidence for the effectiveness of digital health interventions, these have significantly increased because of the restrictions related to COVID-19 [28].

Considerable challenges exist related to how digital health technologies can best be used and integrated to support and facilitate user engagement and provide individualized health information and care [29]. A level of engagement is required to affect a change in health behaviors [30]. Engagement is measured by the extent of use of the intervention (initial log-in and number of activities completed) and the subjective user experience, which is often measured by user satisfaction [31,32].

### Objectives

Despite the growth in the use of the internet among parents to obtain health information and the emergence of digital health interventions to support parents, systematic evaluation of parental engagement with and effectiveness of digital interventions to improve health literacy has not been conducted. The objective of this review is to examine the effect of digital health interventions on health literacy among parents of children aged 0-12 years with a health condition. In this study, health literacy includes the evaluation of parental engagement (use and satisfaction) with digital health interventions, the effect of these interventions on parental health knowledge and health behavior, and the subsequent impact on child health outcomes.

## Methods

This review followed the Joanna Briggs Institute (JBI) methodology for systematic reviews [33] and was conducted according to the registered a priori PROSPERO (International Prospective Register of Systematic Reviews) protocol CRD42020192386. Qualitative and quantitative studies were included as a broad search was required to address the complex health system–related questions [34].

### Inclusion Criteria

#### Participants

This review considered studies that included parents or primary caregivers of children aged 0-12 years with a health condition. Health conditions could be an acute or a chronic disease, diagnosis, or condition. Studies involving children aged ≥12 years were excluded, as children aged ≥12 years are more likely and encouraged to take more responsibility for their health care. Studies were also excluded if health-related information was directed to the child, if the child was healthy, or if the primary users of the digital health intervention were health professionals such as medical staff, nursing staff, health care management, and administrators or researchers. Studies focusing on health promotion or disease prevention (eg, increased knowledge of obesity, physical activity, and smoking) were excluded.

#### Intervention

This review considered studies that examined any digital health intervention that aimed to improve health literacy among parents. Interventions could focus on communication (eg, web-based platforms, mobile apps, videoconferencing, and SMS text messaging), education (eg, videos, web-based platforms, mobile apps, and interactive training), or a combination of communication and education interventions. Interventions that targeted the child or clinician or were delivered directly (eg, face to face by health care professionals) to the parent were excluded.

#### Context

This review considered studies that examined the use of digital health interventions in both the home and hospital setting.

#### Outcomes

This review considered studies that included an increase or decrease in health literacy defined by health knowledge or health behavior [5,30]. The review also considered the following outcomes: changes in the child’s health outcome and engagement with the digital health intervention, including (1) use, that is, *logging into* the web-based platform, *continued use of* the digital health intervention, amount and duration of access, and type of information accessed; or (2) satisfaction with the digital health intervention measured, for example, by attention, interest, usefulness, and perceived benefits through survey items [31,32].

### Search Strategy

The following databases were searched for existing systematic reviews on this topic: CINAHL, MEDLINE, PROSPERO, JBI Database of Systematic Reviews and Implementation Reports, and Cochrane Library Database of Systematic Reviews, and no reviews were located on the specific topic. The base search strategy was developed in CINAHL, and additional adapted searches were run on CINAHL, MEDLINE, and PsycINFO and hand searched in Google Scholar. The search strategy was developed in collaboration with a research librarian to identify articles examining the health literacy of parents of children with a health condition. The key terms were *health literacy* or *health behavior* or *health education* or *health information* and *digital health* or *mobile health* or *electronic health*. The initial search was undertaken in June 2020 and updated in April 2021. The search was limited to papers published in English between January 2010 and April 2021, as there have been constant developments and improvements in digital health technology over the past 10 years. The complete search strategy is shown in Multimedia Appendix 1. The reference list of all the included studies was reviewed to identify any relevant papers not found in the electronic search. Gray literature sources including OpenGrey, ProQuest Dissertation and Theses, Google, and Google Scholar were also searched to identify unpublished studies.

### Screening of Articles

After removing duplicates using Endnote (Clarivate), the web-based tool Rayyan (Rayyan Systems Inc) was used to screen the articles [35]. Titles and abstracts were reviewed independently by 2 authors (EM and SR) to determine if they met the inclusion criteria. Any disagreement regarding eligibility was resolved through discussion with a third author (DA). The selected articles were reviewed in full text by the same authors.

### Assessment of Methodological Quality

The quality of the screened studies was critically appraised independently by 2 reviewers (EM and SR) using the Mixed Methods Appraisal Tool (MMAT) version 2018 [36]. The MMAT was selected in preference to the JBI critical appraisal tool as the MMAT was developed to appraise studies that combine qualitative, quantitative, and mixed methods studies.

### Data Extraction

Data extraction was undertaken by the second author (SR) and reviewed by the first author (EM) and modeled on the standardized data extraction tool from the JBI [33]. The extracted data included specific details about the study setting and context; phenomena of interest (health literacy); study design; sampling of participants, sample size, and characteristics of the study sample; specific details about the interventions; and outcomes of significance to the review question. All data were extracted following a thorough, complete reading of the text to identify qualitative and quantitative findings relevant to the objectives and questions of the review.

### Data Synthesis

Owing to differences in reported quantitative data and the small number of studies, meta-analysis was not possible. The findings are presented in a narrative form [37], including tables and figures to aid in data presentation.

## Results

### Study Inclusion

**Figure 1:**
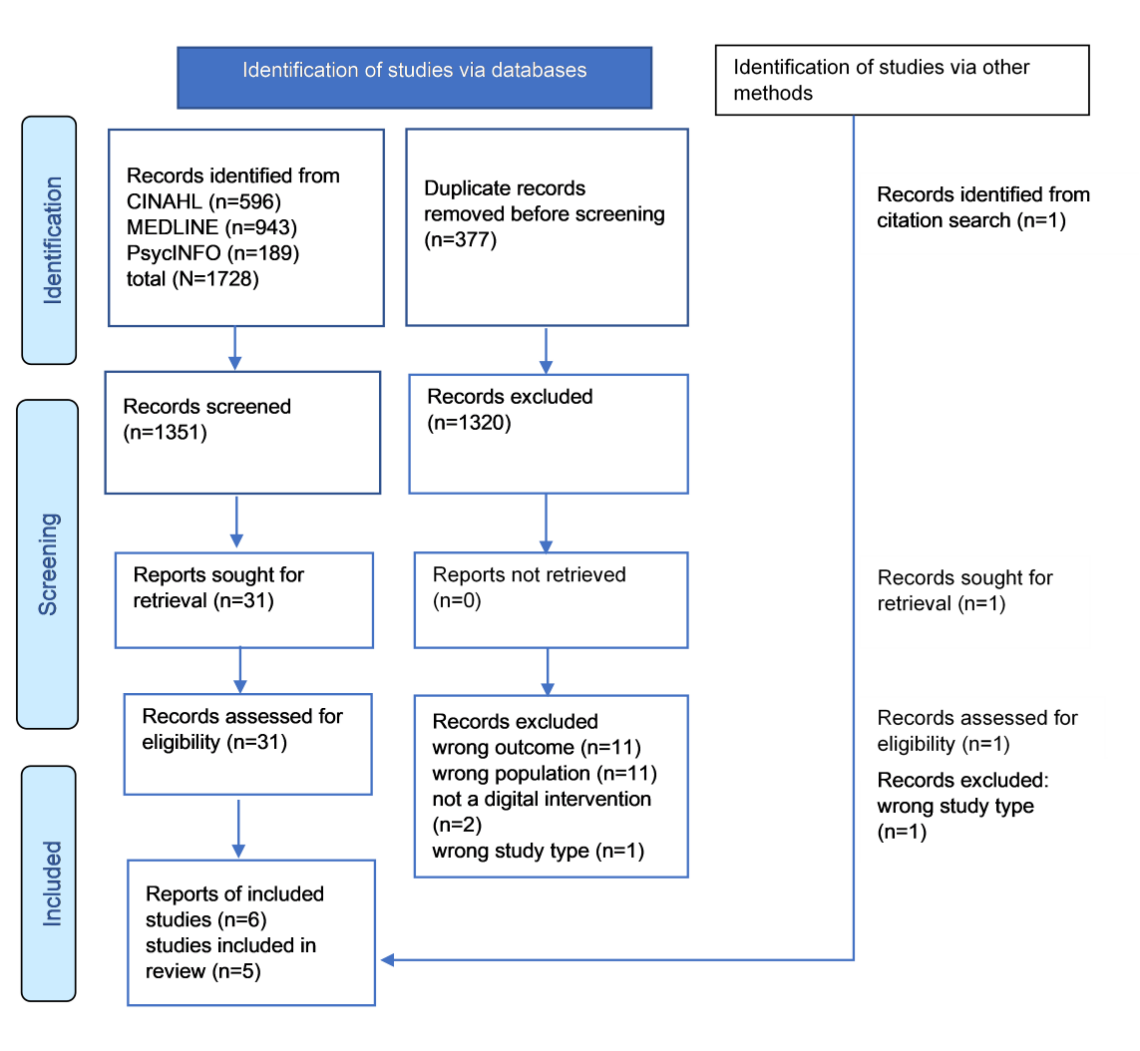
PRISMA (Preferred Reporting Items for Systematic Reviews and Meta-Analyses) flowchart of the study selection and inclusion process.

In total, 1728 references were identified using the search terms. The addition of secondary searches of reference lists and searches of gray literature resulted in the identification of another reference. The exclusion of duplicates resulted in 1351 references, of which 1320 (97.71%) were excluded after the title and abstract screening. The remaining 31 references were retrieved in full text. Of the 31 papers, 25 (81%) were excluded (Multimedia Appendix 2 [38-62]), resulting in 6 (19%) papers eligible for inclusion (Figure 1) [63-68].

### Methodological Quality

A total of 2 authors (EM and SR) independently appraised the 6 articles that met the inclusion criteria for methodological quality. Table 1 summarizes the questions answered in the MMAT, with 1 study excluded as it did not meet the essential screening criteria questions [36,68]. The methods varied across the remaining 5 studies. Of the 5 studies, 2 (40%) studies used mixed methods [63,64] and 3 (60%) studies used a quasi-experimental design (quantitative nonrandomized) [65-67].

**Table 1:** Assessment of methodological quality with the Mixed Methods Appraisal Tool version 2018 [36].

			Blatz et al [63]		Fiks et al [64]		Kobak et al [65]		McGarry et al [66]		Ruiz-Baqués et al [67]		Slater et al [68]
**Screening questions**													
	Are there clear research questions?	Yes		Yes		Yes		Yes		Yes		Unclear	
	Do the collected data allow to address the research questions?	Yes		Yes		Yes		Yes		Yes		Unclear	
**Quantitative descriptive**													
	Is the sampling strategy relevant to address the research question?	Yes		Yes		N/A^a^		N/A		N/A		N/A	
	Is the sample representative of the target population?	Yes		Yes		N/A		N/A		N/A		N/A	
	Are the measurements appropriate?	No		Yes		N/A		N/A		N/A		N/A	
	Is the risk of nonresponse bias low?	Yes		Yes		N/A		N/A		N/A		N/A	
	Is the statistical analysis appropriate to answer the research question?	Yes		Yes		N/A		N/A		N/A		N/A	
**Quantitative nonrandomized**													
	Are the participants representative of the target population?	N/A		N/A		No		No		Yes		N/A	
	Are measurements appropriate regarding both the outcome and intervention (or exposure)?	N/A		N/A		Yes		Yes		No		N/A	
	Are there complete outcome data?	N/A		N/A		Yes		No		Yes		N/A	
	Are the confounders accounted for in the design and analysis?	N/A		N/A		Yes		Yes		Yes		N/A	
	During the study period, was the intervention administered (or exposure occurred) as intended?	N/A		N/A		Yes		Yes		Yes		N/A	
	Quantitative score, n (%)	4 (80)		5 (100)		4 (80)		3 (60)		4 (80)		N/A	
**Qualitative**													
	Is the qualitative approach appropriate to answer the research question?	Yes		Yes		N/A		N/A		N/A		N/A	
	Are the qualitative data collection methods adequate to address the research question?	No		No		N/A		N/A		N/A		N/A	
	Are the findings adequately derived from the data?	No		No		N/A		N/A		N/A		N/A	
	Is the interpretation of results sufficiently substantiated by the data?	No		No		N/A		N/A		N/A		N/A	
	Is there coherence between qualitative data sources, collection, analysis, and interpretation?	No		No		N/A		N/A		N/A		N/A	
	Qualitative score, n (%)	1 (20)^b^		1 (20)^b^		N/A		N/A		N/A		N/A	

^a^N/A: not applicable.

^b^Only the quantitative part was used in the review as the qualitative information provided was minimal.

The quality of the 60% (3/5) quasi-experimental studies was moderate, with a 60% to 80% score [65-67], as was the quality of the quantitative component of the mixed methods studies, which was moderate to good with an 80% to 100% score [63,64]. The quality of the qualitative component of the 40% (2/5) mixed methods studies was poor (20%) and was therefore excluded [63,64].

Although the 5 included studies met the quality criteria, biases were noted. A mixed methods study [63] and a quasi-experimental study [67] used nonvalidated measurement tools. In 67% (2/3) of the quasi-experimental studies, it was unclear if the participants were representative of the population, as a large proportion of participants were White [65] or college-educated [66]. McGarry et al [66] did not provide complete outcome data, with a high rate of participants not completing the program and not providing a rationale for why they dropped out of the program [66]. None of the 5 studies were randomized or had an independent control group, limiting the ability to quantify the effect of the digital health intervention and limit the confounding factors. Of the 5 studies, 3 (60%) had small sample sizes, with not more than 30 participants in each study receiving the digital health intervention [63,65,66]. Of these 3 studies, 2 (67%) were pilot studies [65,66].

### Characteristics of the Studies

Of the 5 studies, 2 (40%) used a descriptive longitudinal design with repeated measures [63,64] and 3 (60%) used a pretest–posttest design [65-67]. Of the 5 studies identified as meeting the selection criteria, 1 (20%) study was implemented in a hospital [63], 2 (40%) in outpatient clinics [64,65], and 2 (40%) in the community [66,67]. Of the 5 studies, 4 (80%) were conducted in the United States [63-66] and 1 (20%) study in Spain [67]. More detailed information about the study characteristics is provided in Multimedia Appendix 3 [63-67].

### Description of the Participants

The sample size varied across the 5 studies, with 3 (60%) studies having <30 participants [63,65,66] and 2 (40%) studies with >200 participants [64,67]. Most participants were mothers, ranging from 73% [66] to 100% of the sample in each study [63]. The mean age of participants in each study varied; the lowest mean age was 28.6 years [63], and the highest was 37.5 years [64]. A higher percentage of male children living with health conditions was represented in the studies, ranging from 54.5% [64] to 73% [66] (Multimedia Appendix 3).

### Description of the Interventions

All the 5 digital interventions used web-based portals, with access limited to the participants only (ie, access was not open to the general public). The interventions targeted a variety of health conditions: infants born preterm [63], asthma [64], autism spectrum disorder [65,66], and food allergy [67]. All 5 study interventions included an educational component, with 4 (80%) studies including additional interactive communication components (electronic recording of times and volumes of breastmilk expression, a patient portal to interact with health care providers [64], use of videos, web-based training of parents to promote the child’s communication skills [66], and web-based discussion forums [67]; Multimedia Appendix 4 [63-67]).

### Parent-Related Outcomes

**Figure 2:**
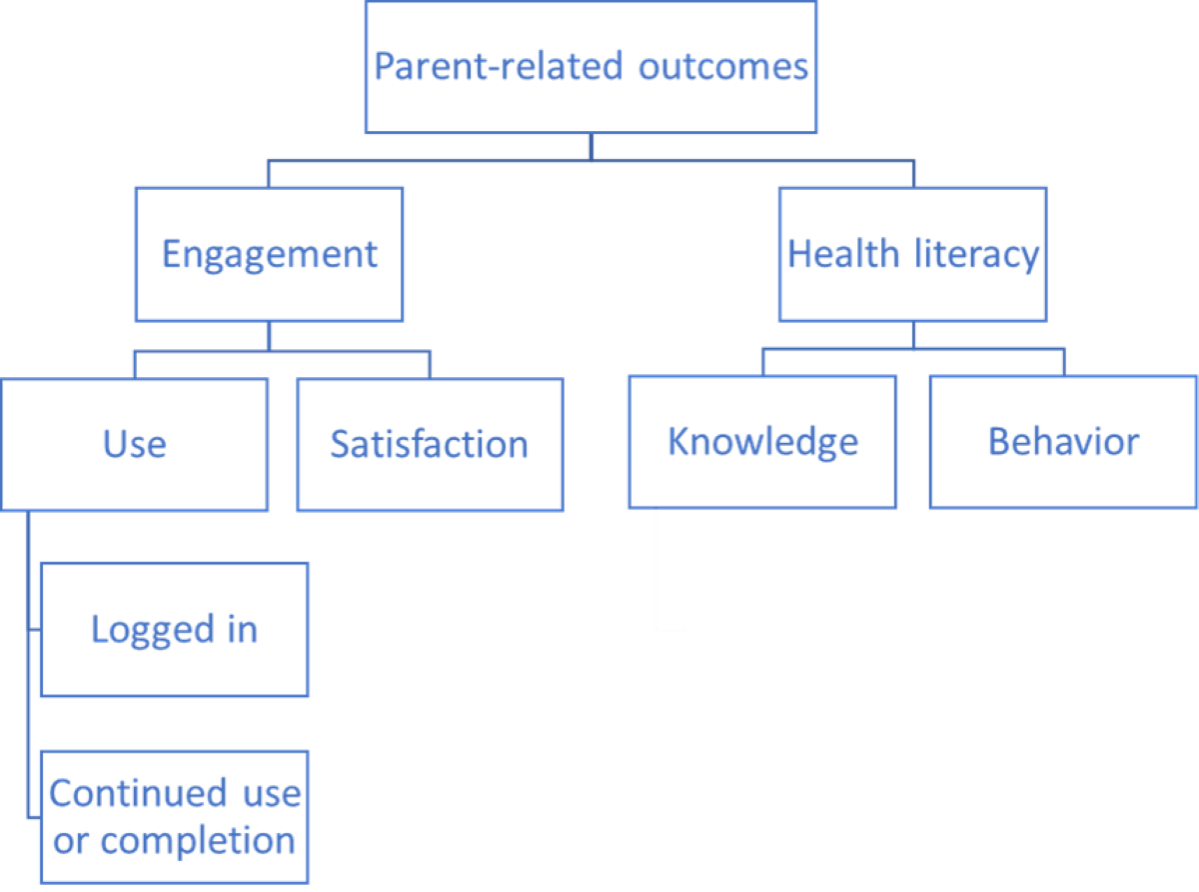
Parent-related outcomes extracted as engagement and health literacy, including subcontent.

Parent-related outcomes were extracted as engagement (use and satisfaction) and health literacy (health knowledge and health behavior; Figure 2).

#### Engagement

All studies reported on engagement, although the definitions of engagement and levels of engagement varied across the studies. Additional details are outlined in Multimedia Appendix 4 and further described in the following sections.

##### Use

Approximately 80% (4/5) of the studies reported the number of parents who were invited to participate [63,64,66,67]. Invitees logging into the website ranged from 2.6% of eligible parents [64] to 100% [66] (Table 2). Most participants (67%-100%) who logged into the website accessed the digital intervention at least once [63-67]. Users who continued to use the intervention or completed the digital health intervention ranged from 37% of parents participating in the Pivotal Response Treatment (PRT) program [66] to 100% in the Lactation Log Plus website [63] and the web-based tutorial, which focused on communication with children with autism spectrum disorder [65] (Table 2).

**Table 2:** Number and percentage of parents approached to participate, logged into the website, and accessed the digital intervention (use).

Study	Approached to participate, N	Logged into the website, n (%)	Accessed the intervention, n (%)	Continued use or completed the intervention, n (%)
Blatz et al [63]	20	18 (90)	13 (100)	13 (100)
Fiks et al [64]	9133	237 (2.59)	237 (100)	156 (65.8)
Kobak et al [65]	—^a^	23 (—)	23 (100)	23 (100)
McGarry et al [66]	51	30 (59)	30 (100)	11 (37)
Ruiz-Baqués et al [67]	277	207 (74.5)	139 (67.1)	130 (62.8)

^a^Not provided.

##### Satisfaction

Of the 5 studies, 3 (60%) measured satisfaction with digital health interventions. Tools used to measure satisfaction varied, with high satisfaction identified in all 3 studies [65-67].

Kobak et al [65] used the System Usability Scale to measure satisfaction with the technical parts of the web-based version of the Enhancing Interaction Program. This is a validated 10-item scale ranging from 0 to 100. The mean score in this study was 85 (SD 17), which corresponds to a score of excellent. They also used the User Satisfaction Questionnaire to evaluate the clinical content of the web-based program. This scale ranges from 15 to 60 and has shown good internal consistency. The mean score in this study was 54.5 (SD 5.9). McGarry et al [66] used Social Validity Measures to assess parents’ satisfaction with the PRT program. Parents were asked to respond to a variety of statements using a scale from 0 (strongly disagree) to 5 (strongly agree). Over 90% of the parents agreed that the course was well-written and organized. Ruiz-Baqués et al [67] used a 5-item Likert scale to assess satisfaction with the educational program. The scale ranged from 0 (not at all) to 10 (great deal) points. The mean score in this study was 8.78.

#### Health Literacy

All studies reported on health literacy, either as a change in health knowledge or health behavior. Additional details are outlined in Multimedia Appendix 4 and further described in the following sections.

##### Health Knowledge

Improvement in parental knowledge was identified in 40% (2/5) of the studies [65,67]. Kobak et al [65] used a questionnaire to measure changes in knowledge before and after a web-based intervention program for autism spectrum disorder and found an increase in the mean number of correct items from 12.6 to 20.4 (*P*<.001); 79% of parents scored ≥80% after taking the tutorial compared with 8% before taking the tutorial. Ruiz-Baqués et al [67] measured the changes in knowledge before and after a web-based intervention program for food allergy and found an improvement in 15 out of 30 questionnaire items and a significant improvement (*P*<.001) in 8 items. Improvement was more frequent in the *general knowledge and clinical aspects* domain than in the *daily life with food allergy* domain. No study reported on the time elapsed between the end of the intervention and the knowledge test.

##### Health Behavior

Of the 5 studies, improvement in behavior was identified in 3 (60%) studies [63,64,66]. Blatz et al [63] found that a website that included breast milk information and a milk diary helped participants pump milk and sustain milk supply. Of the 13 participants, 2 (15%) felt that the milk log website helped to pump milk a great deal, 5 (38%) felt that it somewhat helped, and 6 (46%) felt that the website log did not help them pump breast milk. Furthermore, of the 13 participants, 2 (15%) felt that the milk log website helped maintain milk supply a great deal, 3 (23%) felt that it somewhat helped, and 8 (62%) felt that the website log did not help them maintain it.

Fiks et al [64] found increased medication refills and asthma-related medical visits among parents of children with uncontrolled asthma. Of the 76 children with uncontrolled asthma after the first survey, 20 (67%) had a medication change or refill within 30 days of survey completion, and 21 (28%) had an asthma-related primary care visit within 30 days. The results represent a significant increase in medication changes or refills and asthma-related visits when compared with the same period in the prior year for each child (14% increase in medication changes, 95% CI 2%-27%, and 16% increase in visits, 95% CI 3%-28%).

McGarry et al [66] found that parents who completed the Autism PRT program were successfully able to learn and implement the strategies. Parents submitted videos of parent-child interactions, which were coded for the fidelity of implementation and social communication behaviors. Parent’s treatment fidelity improved from baseline (mean 65.34%, SD 18.04%) to week 5 (mean 90.13%, SD 7.20%; *P*<.001). At baseline, of the 11 parents, 1 (9%) met the fidelity of implementation, with ≥80% fidelity score. By the end of the program, 10 parents met the fidelity of implementation, whereas 1 parent approached fidelity with a 75% fidelity score.

### Child Health Outcomes

Of the 5 studies, 1 study (20%) reported on child health outcomes. McGarry et al [66] demonstrated increased communication behavior among children with autism spectrum disorder. There was an improvement from baseline to week 5 in children’s vocalization (*P*=.05), eye contact (*P*=.03), and positive affect (*P*<.001).

## Discussion

### Principal Findings

With only 5 intervention studies identified in this systematic review, it is clear that few digital health interventions have been developed to improve health literacy among parents of children aged 0 to 12 years living with a health condition. Of the 5 studies, 4 (80%) were published in the past 4 years, suggesting that digital health interventions to improve health literacy are an emerging area of research. The use of digital health interventions in clinical practice has also increased because of the COVID-19 pandemic [25].

The 5 studies reported parent-related outcomes, with a focus on engagement with digital health interventions. Despite the low number of studies, digital health interventions to improve health literacy appear acceptable and useful among parents. After parents logged into a website and enrolled in the digital health intervention, >60% of parents demonstrated continued engagement with the program [63-65,67], with 1 exception [66]. The study by McGarry et al [66] had the lowest rate of continued use. The reason could be a rather demanding program where the parents had to submit a video after each web-based lesson, capturing how they used their new information with the child. Submission of a video was a prerequisite for continuing with the intervention. In another study, <3% of the parents who were approached logged into the intervention platform [64]. However, in the same study, approximately 66% of the parents continued to use the portal once logged in [64], implying that parents who initiate engagement are also likely to remain engaged. A systematic review studying the factors affecting engagement and recruitment to digital health interventions suggests that people struggle to make sense of digital health interventions and recommends raising the profile for digital health products to make people more aware of them [69]. It is possible that the current pandemic, which has increased the use of digital health care, has raised the awareness of digital interventions.

Improvement in parental health literacy, including either knowledge [65,67] or behavior change [63,64,66], indicated positive results, which are important when parents are responsible for their children’s health care and well-being [1]. However, none of the included studies used a validated instrument developed to measure general health literacy, such as Rapid Estimate of Adult Literacy in Medicine or Test of Functional Health Literacy in Adults [70]. Instead, both Kobak et al [65] and Ruiz-Baqués et al [67] measured specific knowledge targeting autism spectrum disorder and food allergy, respectively. Disease-specific knowledge has proved to be of greater importance in affecting health behavior change than general health literacy [71]. It is also suggested that general health literacy can be a prerequisite for disease-specific knowledge [71]. Health literacy is a complex concept in which knowledge is important and can influence health behavior and child health outcomes [5]. For parents responsible for their children’s basic care and specific care related to their health condition, it is essential to have both general health literacy and disease-specific knowledge. However, few studies have evaluated the effect of digital health interventions on either parents’ general health literacy or disease-specific knowledge. In times of a pandemic, when access to physical consultations with health care providers has been affected, digital health interventions to increase parents’ knowledge and behavior may be of utmost importance for the child’s health.

Health literacy increases the parent’s capacity to engage in and take responsibility for their child’s health care [2], both of which are associated with improved child health outcomes. Only 20% (1/5) of the studies reported on a change in outcomes [66]. McGarry et al [66] reported positive changes in children’s communicative behavior at the same time as the parent’s treatment fidelity improved significantly. These are promising results showing that increased disease-specific knowledge in parents can positively affect child health outcomes; however, more studies are necessary to prove this hypothesis. Therefore, it is essential to report on potential improvements in the child’s health status in future studies to evaluate the impact of increased health literacy in parents.

No randomized controlled studies were included in this review. Of the 5 studies, 2 (40%) were presented as mixed methods studies [63,64]. Unfortunately, the qualitative part of these studies was assessed as having low methodological quality. The only qualitative study included following full-text review was subsequently excluded after critical appraisal [68]. The 3 quantitative studies that evaluated satisfaction reported high satisfaction levels with the digital health intervention [65-67]. However, these studies all used different tools to measure satisfaction, thus limiting the aggregation and synthesis of data. Qualitative studies could help to develop an understanding of why some parents decide to initiate engagement with digital health interventions and what factors contribute to their continued use.

### Limitations of the Review

Although this review was systematic, the findings must be interpreted with caution. The number of studies identified was small, homogeneity among the studies was limited, and none of the included studies used a true comparison (ie, control group). Several factors may have influenced the outcome of the digital health interventions, including the implementation methodology, limited responses and participation rates, encouragement by health care providers, and participants’ characteristics. Owing to the small number of included studies and missing data on participants’ characteristics, the influence of potential covariates could not be further evaluated. The variation in methodological design, including differences in outcomes and few comparators, limited the authors’ ability to conduct a meta-analysis. It should also be considered that only studies published in English were included in this review. Despite these limitations, the favorable results across studies suggest that further evaluations of the benefits of digital health interventions should be undertaken.

### Conclusions

This review has shown the potential of digital health interventions to improve health knowledge and health behavior among parents of children aged 0-12 years with a health condition. Of the 5 included studies, 4 (80%) were published in the past 4 years, indicating that digital health interventions aimed at improving health literacy are a developing research area. Future studies should include qualitative studies and studies with randomized samples to more fully understand the potential of digital health interventions to increase parent health literacy.

## Appendix

Multimedia Appendix 1 Search strategy.

Multimedia Appendix 2 Excluded studies.

Multimedia Appendix 3 Descriptive information.

Multimedia Appendix 4 Description of interventions and outcomes.

## Abbreviations

JBI: Joanna Briggs Institute
MMAT: Mixed Methods Appraisal Tool
PROSPERO: International Prospective Register of Systematic Reviews
PRT: Pivotal Response Treatment

## References

[1] Boshoff K, Gibbs D, Phillips RL, Wiles L, Porter L (2016). Parents' voices: 'why and how we advocate'. A meta-synthesis of parents' experiences of advocating for their child with autism spectrum disorder. Child Care Health Dev.

[2] Aarthun A, Øymar KA, Akerjordet K (2018). How health professionals facilitate parents' involvement in decision-making at the hospital: a parental perspective. J Child Health Care.

[3] Smith J, Swallow V, Coyne I (2015). Involving parents in managing their child's long-term condition-a concept synthesis of family-centered care and partnership-in-care. J Pediatr Nurs.

[4] Smith J, Kendal S (2018). Parents' and health professionals' views of collaboration in the management of childhood long-term conditions. J Pediatr Nurs.

[5] Sørensen K, Van den Broucke S, Fullam J, Doyle G, Pelikan J, Slonska Z, Brand H, (HLS-EU) Consortium Health Literacy Project European (2012). Health literacy and public health: a systematic review and integration of definitions and models. BMC Public Health.

[6] de Buhr E, Tannen A (2020). Parental health literacy and health knowledge, behaviours and outcomes in children: a cross-sectional survey. BMC Public Health.

[7] Berkman N, Sheridan S, Donahue K, Halpern D, Viera A, Crotty K, Holland A, Brasure M, Lohr KN, Harden E, Tant E, Wallace I, Viswanathan M (2011). Health literacy interventions and outcomes: an updated systematic review. Evid Rep Technol Assess (Full Rep).

[8] DeWalt DA, Hink A (2009). Health literacy and child health outcomes: a systematic review of the literature. Pediatrics.

[9] Sanders LM, Federico S, Klass P, Abrams MA, Dreyer B (2009). Literacy and child health: a systematic review. Arch Pediatr Adolesc Med.

[10] Lenahan JL, McCarthy DM, Davis TC, Curtis LM, Serper M, Wolf MS (2013). A drug by any other name: patients' ability to identify medication regimens and its association with adherence and health outcomes. J Health Commun.

[11] Yin HS, Mendelsohn AL, Wolf MS, Parker RM, Fierman A, van Schaick L, Bazan IS, Kline MD, Dreyer BP (2010). Parents' medication administration errors: role of dosing instruments and health literacy. Arch Pediatr Adolesc Med.

[12] Yin HS, Dreyer BP, Foltin G, van Schaick L, Mendelsohn AL (2007). Association of low caregiver health literacy with reported use of nonstandardized dosing instruments and lack of knowledge of weight-based dosing. Ambul Pediatr.

[13] Harrington KF, Zhang B, Magruder T, Bailey WC, Gerald LB (2015). The impact of parent's health literacy on pediatric asthma outcomes. Pediatr Allergy Immunol Pulmonol.

[14] Shone LP, Conn KM, Sanders L, Halterman JS (2009). The role of parent health literacy among urban children with persistent asthma. Patient Educ Couns.

[15] Morrison AK, Glick A, Yin HS (2019). Health literacy: implications for child health. Pediatr Rev.

[16] Tzeng Y, Chiang B, Chen Y, Gau B (2018). Health literacy in children with asthma: a systematic review. Pediatr Neonatol.

[17] Pulgarón ER, Sanders LM, Patiño-Fernandez AM, Wile D, Sanchez J, Rothman RL, Delamater AM (2014). Glycemic control in young children with diabetes: the role of parental health literacy. Patient Educ Couns.

[18] Arabiat D, Whitehead L, Foster M, Shields L, Harris L (2018). Parents' experiences of Family Centred Care practices. J Pediatr Nurs.

[19] Cousino MK, Hazen RA (2013). Parenting stress among caregivers of children with chronic illness: a systematic review. J Pediatr Psychol.

[20] Angelhoff C, Edéll-Gustafsson U, Mörelius E (2015). Sleep of parents living with a child receiving hospital-based home care: a phenomenographical study. Nurs Res.

[21] Mörelius E, Hemmingsson H (2014). Parents of children with physical disabilities - perceived health in parents related to the child's sleep problems and need for attention at night. Child Care Health Dev.

[22] Thompson D, Leach M, Smith C, Fereday J, May E (2020). How nurses and other health professionals use learning principles in parent education practice: a scoping review of the literature. Heliyon.

[23] Kepreotes E, Keatinge D, Stone T (2010). The experience of parenting children with chronic health conditions: a new reality. J Nurs Healthc Chronic Illn.

[24] Meyers N, Glick AF, Mendelsohn AL, Parker RM, Sanders LM, Wolf MS, Bailey S, Dreyer BP, Velazquez JJ, Yin HS (2020). Parents' use of technologies for health management: a health literacy perspective. Acad Pediatr.

[25] Kubb C, Foran HM (2020). Online health information seeking by parents for their children: systematic review and agenda for further research. J Med Internet Res.

[26] Jaks R, Baumann I, Juvalta S, Dratva J (2019). Parental digital health information seeking behavior in Switzerland: a cross-sectional study. BMC Public Health.

[27] Shorey S, Chee CY, Ng ED, Lau Y, Dennis C, Chan YH (2019). Evaluation of a technology-based peer-support intervention program for preventing postnatal depression (part 1): randomized controlled trial. J Med Internet Res.

[28] Monaghesh E, Hajizadeh A (2020). The role of telehealth during COVID-19 outbreak: a systematic review based on current evidence. BMC Public Health.

[29] McCabe C, Timmins F (2016). Embracing healthcare technology - What is the way forward for nurse education?. Nurse Educ Pract.

[30] Short CE, DeSmet A, Woods C, Williams SL, Maher C, Middelweerd A, Müller AM, Wark PA, Vandelanotte C, Poppe L, Hingle MD, Crutzen R (2018). Measuring engagement in eHealth and mHealth behavior change interventions: viewpoint of methodologies. J Med Internet Res.

[31] Perski O, Blandford A, West R, Michie S (2017). Conceptualising engagement with digital behaviour change interventions: a systematic review using principles from critical interpretive synthesis. Transl Behav Med.

[32] Yardley L, Spring BJ, Riper H, Morrison LG, Crane DH, Curtis K, Merchant GC, Naughton F, Blandford A (2016). Understanding and promoting effective engagement with digital behavior change interventions. Am J Prev Med.

[33] (2020). JBI manual for evidence synthesis. JBI Global.

[34] Noyes J, Booth A, Moore G, Flemming K, Tunçalp Ö, Shakibazadeh E (2019). Synthesising quantitative and qualitative evidence to inform guidelines on complex interventions: clarifying the purposes, designs and outlining some methods. BMJ Glob Health.

[35] Ouzzani M, Hammady H, Fedorowicz Z, Elmagarmid A (2016). Rayyan-a web and mobile app for systematic reviews. Syst Rev.

[36] Hong QN, Pluye P, Fàbregues S, Bartlett G, Boardman F, Cargo M, Dagenais P, Gagnon M, Griffiths F, Nicolau B, O'Cathain A, Rousseau M, Vedel I (2019). Improving the content validity of the mixed methods appraisal tool: a modified e-Delphi study. J Clin Epidemiol.

[37] Tufanaru C, Munn Z, Aromataris E, Campbell J, Hopp L Chapter 3: Systematic reviews of effectiveness. JBI Manual for Evidence Synthesis.

[38] Armstrong-Heimsoth A, Johnson Ml, McCulley A, Basinger M, Maki K, Davison D (2017). Good googling: a consumer health literacy program empowering parents to find quality health information online. J Consum Health Internet.

[39] Ayre J, Costa DS, McCaffery KJ, Nutbeam D, Muscat DM (2020). Validation of an Australian parenting health literacy skills instrument: the parenting plus skills index. Patient Educ Couns.

[40] Asan O, Scanlon MC, Crotty B, Holden RJ, Flynn KE (2019). Parental perceptions of displayed patient data in a PICU: an example of unintentional empowerment. Pediatr Crit Care Med.

[41] Byczkowski TL, Munafo JK, Britto MT (2014). Family perceptions of the usability and value of chronic disease web-based patient portals. Health Informatics J.

[42] Chau S, Oldman S, Smith SR, Lin CA, Ali S, Duffy VB (2021). Online behavioral screener with tailored obesity prevention messages: application to a pediatric clinical setting. Nutrients.

[43] Chorianopoulou A, Lialiou P, Mechili E-A, Mantas J, Diomidous M (2015). Investigation of the quality and effectiveness of telemedicine in children with diabetes. Stud Health Technol Inform.

[44] Dudovitz R, Teutsch C, Holt K, Herman A (2020). Improving parent oral health literacy in Head Start programs. J Public Health Dent.

[45] Edwards C, Bolling-Walker K, Deupree J (2020). Actionability and usability of a fever management tool for pediatric caregivers. J Contin Educ Nurs.

[46] Fagnano M, Halterman JS, Conn KM, Shone LP (2012). Health literacy and sources of health information for caregivers of urban children with asthma. Clin Pediatr (Phila).

[47] Fauer AJ, Hoodin F, Lalonde L, Errickson J, Runaas L, Churay T, Seyedsalehi S, Warfield C, Chappell G, Brookshire K, Chaar D, Shin JY, Byrd M, Magenau J, Hanauer DA, Choi SW (2019). Impact of a health information technology tool addressing information needs of caregivers of adult and pediatric hematopoietic stem cell transplantation patients. Support Care Cancer.

[48] Gage-Bouchard EA, LaValley S, Mollica M, Beaupin LK (2017). Communication and exchange of specialized health-related support among people with experiential similarity on facebook. Health Commun.

[49] Guðmundsdóttir K, Ala’i-Rosales S, Sigurðardóttir Z (2018). Extending caregiver training via telecommunication for rural Icelandic children with autism. Rural Special Educ Q.

[50] Kaskinen A, Ayeboa-Sallah B, Teivaanmäki T, Wärnhjelm E, Korhonen L, Helve O (2018). Pediatric web-based chat services for caregivers of children: descriptive study. J Med Internet Res.

[51] Liu J, Zheng X, Zhang X, Feng Z, Song M, Lopez V (2020). The evidence and future potential of WeChat in providing support for Chinese parents of pediatric patients undergoing herniorrhaphy. J Transcult Nurs.

[52] Macken AP, Sasaki E, Quinn A, Cullen W, Leddin D, Dunne C, O'Gorman CS (2014). Paediatric diabetes: information-seeking behaviours of families. Ir Med J.

[53] McCarty CA, Zatzick DF, Marcynyszyn LA, Wang J, Hilt R, Jinguji T, Quitiquit C, Chrisman SP, Rivara FP (2021). Effect of collaborative care on persistent postconcussive symptoms in adolescents: a randomized clinical trial. JAMA Netw Open.

[54] McCarty C, Vander Stoep A, Violette H, Myers K (2014). Interventions developed for psychiatric and behavioral treatment in the children’s ADHD telemental health treatment study. J Child Fam Stud.

[55] Meedya S, Win K, Yeatman H, Fahy K, Walton K, Burgess L, McGregor D, Shojaei P, Wheatley E, Halcomb E (2021). Developing and testing a mobile application for breastfeeding support: the Milky Way application. Women Birth.

[56] Price S, Ferisin S, Sharifi M, Steinberg D, Bennett G, Wolin KY, Horan C, Koziol R, Marshall R, Taveras EM (2015). Development and implementation of an interactive text messaging campaign to support behavior change in a childhood obesity randomized controlled trial. J Health Commun.

[57] Ramelet A-S, Fonjallaz B, Rio L, Zoni S, Ballabeni P, Rapin J, Gueniat C, Hofer M (2017). Impact of a nurse led telephone intervention on satisfaction and health outcomes of children with inflammatory rheumatic diseases and their families: a crossover randomized clinical trial. BMC Pediatr.

[58] Saidinejad M, Zorc J (2014). Mobile and web-based education: delivering emergency department discharge and aftercare instructions. Pediatr Emerg Care.

[59] Sharifi M, Dryden EM, Horan CM, Price S, Marshall R, Hacker K, Finkelstein JA, Taveras EM (2013). Leveraging text messaging and mobile technology to support pediatric obesity-related behavior change: a qualitative study using parent focus groups and interviews. J Med Internet Res.

[60] Spratling R, Faulkner MS, Chambers R, Lawrence P, Feinberg I, Hayat MJ (2020). Establishing fidelity for the creating opportunities for personal empowerment: symptom and technology management resources (COPE-STAR) intervention. J Adv Nurs.

[61] Tutar Güven Ş, İşler Dalgiç A, Duman Ö (2020). Evaluation of the efficiency of the web-based epilepsy education program (WEEP) for youth with epilepsy and parents: a randomized controlled trial. Epilepsy Behav.

[62] van der Gugten AC, Uiterwaal CS, Verheij TJ, van der Ent CK (2015). E-health and consultation rates for respiratory illnesses in infants: a randomised clinical trial in primary care. Br J Gen Pract.

[63] Blatz M, Dowling D, Underwood P, Bieda A, Graham G (2017). A password-protected web site for mothers expressing milk for their preterm infants. Adv Neonatal Care.

[64] Fiks AG, DuRivage N, Mayne SL, Finch S, Ross ME, Giacomini K, Suh A, McCarn B, Brandt E, Karavite D, Staton EW, Shone LP, McGoldrick V, Noonan K, Miller D, Lehmann CU, Pace WD, Grundmeier RW (2016). Adoption of a portal for the primary care management of pediatric asthma: a mixed-methods implementation study. J Med Internet Res.

[65] Kobak KA, Stone WL, Wallace E, Warren Z, Swanson A, Robson K (2011). A web-based tutorial for parents of young children with autism: results from a pilot study. Telemed J E Health.

[66] McGarry E, Vernon T, Baktha A (2020). Brief report: a pilot online pivotal response treatment training program for parents of toddlers with autism spectrum disorder. J Autism Dev Disord.

[67] Ruiz-Baqués A, Contreras-Porta J, Marques-Mejías M, Cárdenas Rebollo JM, Capel Torres F, Ariño Pla MN, Zorrozua Santisteban A, Chivato T (2018). Evaluation of an online educational program for parents and caregivers of children with food allergies. J Investig Allergol Clin Immunol.

[68] Slater PJ, Fielden PE, Bradford NK (2018). The oncology family app: providing information and support for families caring for their child with cancer. J Pediatr Oncol Nurs.

[69] O'Connor S, Hanlon P, O'Donnell CA, Garcia S, Glanville J, Mair FS (2016). Understanding factors affecting patient and public engagement and recruitment to digital health interventions: a systematic review of qualitative studies. BMC Med Inform Decis Mak.

[70] Chew LD, Griffin JM, Partin MR, Noorbaloochi S, Grill JP, Snyder A, Bradley KA, Nugent SM, Baines AD, Vanryn M (2008). Validation of screening questions for limited health literacy in a large VA outpatient population. J Gen Intern Med.

[71] Schrauben SJ, Cavanaugh KL, Fagerlin A, Ikizler TA, Ricardo AC, Eneanya ND, Nunes JW (2020). The relationship of disease-specific knowledge and health literacy with the uptake of self-care behaviors in CKD. Kidney Int Rep.

