# Supplementary material for: Digital Interventions to Improve Health Literacy Among Parents of Children Aged 0 to 12 Years With a Health Condition: Systematic Review
Source: J Med Internet Res. 2021 Dec 22;23(12):e31665. doi: 10.2196/31665 (PMC8734927; doi:10.2196/31665)
Supplement: Multimedia Appendix 1 [file jmir_v23i12e31665_app1.docx]

Appendix 1 Search strategi

CINAHL, MEDLINE, PSYCHINFO searched on 15 June 2020 and 21 April 2021

| Search | Query |
| --- | --- |
| #1 | "parent*" OR (MH "Mothers") OR "mother*" OR "father*" OR "mum*" OR "dad*" OR (MH "Fathers") OR (MH "Parents") OR "caregiver*" AND (MH "Child") OR "caregiver*" OR (MH "Child") |
| #2 | AND |
| #3 | "help seeking behaviour” OR (MH "Health Behavior") " OR "health seeking behavior OR "health seeking behaviour" OR "health seeking behaviour" OR (MH "Health Information”) OR "health information" OR (MH "Health Literacy") OR "health literacy" OR (MH "Help Seeking Behavior") OR (MH "Health Education") OR (MH "Information Seeking Behavior") OR "health education"OR "help seeking behavior" OR "health behaviour" OR "health behavior" |
| #4 | AND |
| #5 | "electronic health" OR "e-health" OR "ehealth" OR "digital medicine*" OR "digital health*" OR "digital health intervention" OR (MH "Telemedicine") OR (MH "Telemedicine") OR "mobile app*"OR (MH "Health Literacy") OR (MH "Mobile Applications") OR "mobile health" OR "m-health" OR "mhealth" OR "telehealth" OR "telecare" OR (MH "Email") OR "mobile phone" "text messaging" OR (MH "Text Messaging")OR (MH "Cellular Phone") OR (MH "Smartphone") |
| LIMITED | Published 2010 onwards; English Language |
