# Supplementary material for: Digital Interventions to Improve Health Literacy Among Parents of Children Aged 0 to 12 Years With a Health Condition: Systematic Review
Source: J Med Internet Res. 2021 Dec 22;23(12):e31665. doi: 10.2196/31665 (PMC8734927; doi:10.2196/31665)
Supplement: Multimedia Appendix 2 [file jmir_v23i12e31665_app2.docx]

Appendix 2 List of excluded studies and reason for exclusion

| Reference | Excluded article | Reason for exclusion |
| --- | --- | --- |
| 38 | Armstrong-Heimsoth et al. 2017. | Wrong population; included adults |
| 39 | Ayre et al. 2020. | Wrong outcome; health literacy not included |
| 40 | Asan et al. 2019. | Wrong population; children > 12 years included |
| 41 | Byczkowski et al. 2014. | Wrong population; children > 12 years included |
| 42 | Chau et al. 2021. | Wrong population; health promotion is not a short-term or long-term health condition. |
| 43 | Chorianopoulou et al. 2015. | Wrong outcome; health literacy not included |
| 44 | Dudovitz et al. 2020. | Wrong population; health promotion is not a short-term or long-term health condition. |
| 45 | Edwards et al. 2020. | Wrong intervention; not digital |
| 46 | Fagnano et al. 2012. | Wrong outcome; health literacy not included |
| 47 | Fauer et al. 2019. | Wrong outcome; health literacy not included |
| 48 | Gage-Bouchard eet al. 2017. | Wrong population; children > 12 years included |
| 49 | Guðmundsdóttir et al. 2018. | Wrong population; children > 12 years included |
| 50 | Kaskinen et al. 2018. | Wrong outcome; health literacy not included |
| 51 | Liu et al. 2020. | Wrong outcome, health literacy not included |
| 52 | Macken et al. 2014. | Wrong population; children > 12 years included |
| 53 | McCarty et al. 2021. | Wrong population; children > 12 years included |
| 54 | McCarty et al. 2014. | Wrong outcome; health literacy not included |
| 55 | Meedya et al. 2021. | Wrong population; healthy children |
| 56 | Price et al. 2015. | Wrong outcome; health literacy not included |
| 57 | Ramelet et al. 2017. | Wrong intervention; not digital |
| 58 | Saidinejad & Zorc. 2014. | Wrong study type; discussion paper |
| 59 | Sharifi et al. 2013. | Wrong outcome; intervention not implemented |
| 60 | Spratling et al. 2020. | Wrong outcome; health literacy not included |
| 61 | Tutar Güven et al. 2020. | Wrong population; children aged over children  > 12 years included |
| 62 | van der Gugten et al. 2015. | Wrong outcome; health literacy not included |
