# Supplementary material for: Digital Interventions to Improve Health Literacy Among Parents of Children Aged 0 to 12 Years With a Health Condition: Systematic Review
Source: J Med Internet Res. 2021 Dec 22;23(12):e31665. doi: 10.2196/31665 (PMC8734927; doi:10.2196/31665)
Supplement: Multimedia Appendix 3 [file jmir_v23i12e31665_app3.docx]

| **Author**  **Year** | **Health condition** | **Study type** | **Aims** | | **Setting** | **Participant characteristics** |
| --- | --- | --- | --- | --- | --- | --- |
| **Blatz et al.**  **2017 [63]** | Premature birth | Mixed methods. Study design:  descriptive longitudinal study with qualitative comments. | | To explore the efficacy of the use of a password-protected website designed exclusively for mothers of preterm infants hospitalized in the neonatal intensive care unit who elect to breastfeed and/or provide breast milk. | ***Country:*** USA  ***Recruitment area*:** Hospital based  neonatal intensive care unit and/or transitional care unit in a large Midwestern urban academic medical centre. | ***Parents***  **Age:** x̄=28.6, SD=6.2 years (range 20-39).  **Sex:** Female 100%.  **Ethnicity:**  40% Caucasian, 55% African American, 5% other  **Education:**  50% High school or less  50% College or higher  ***Babies***  **Age:** x̄=2.4, SD=0.9 days (range 1-5).  **Sex:** 7 females, 14 males. |
| **Fiks et al.**  **2016 [64]** | Asthma | Mixed methods study design: descriptive longitudinal design with  monthly survey and semi-structured interviews. | | To evaluate a portal in paediatric primary care to facilitate communication between families and clinicians regarding treatment concerns and goals, asthma symptoms, medication use, and side effects. | ***Country:*** USA  ***Recruitment area*** Twenty primary care practices were enrolled from 2 practice-based research networks across 11 states. | ***Parents***  **Age:** Parents x̄=37.5, SD=5.8.  **Sex:** Female 96.2%.  **Ethnicity:**  62.4% Caucasian, 28.7% African American, 1.7% Asian, 7.2% Hispanic/Latino.  **Education:**  14.3% High school or less  85.7% College or higher.  ***Children***  **Age:** 74% aged 6-9 years and 26% aged 10-12 years.  **Sex:** 54.5% male, 45.5% female. |

| **Kobak et al. 2011 [65]** | Autism Spectrum Disorder (ASD) | Quasi-experimental design: pre-test post-test design. | To evaluate the efficacy and user satisfaction of a web-based version of the Enhancing Interactions program that teaches parents how to incorporate evidence-based intervention techniques in their daily interactions. | ***Country:*** USA  ***Recruitment area:*** Treatment and Research Institute for ASD (TRIAD), Vanderbilt University, Nashville. | ***Parents***  **Age:** x̄=33.7, SD=6.4 (range 24–51).  **Sex**: Not provided.  **Ethnicity:** 74% Caucasian, 22% African American, 4% other.  **Education:** Not provided.  ***Children***  **Age:** 18 months to 6 years.  **Sex: N**ot provided. |
| --- | --- | --- | --- | --- | --- |
| **McGarry et al. 2020 [66]** | Autism Spectrum Disorder (ASD) | Quasi-experimental design: pre-test post-test design. | To assess the feasibility and acceptability of the online parent training program for toddlers with ASD (Pivotal Response Treatment (PRT)) intervention and to examine parent mastery of PRT procedures and their perceptions of child behaviour change. | ***Country:*** USA  ***Recruitment area***  Parents of children with ASD were recruited from social media advertisements, website listings, and referrals from other professionals and agencies. | ***Parents***  **Age:** Not provided.  **Sex**: Female 72.7%.  **Ethnicity**: 28% Caucasian, 18% Asian, 36% Hispanic/Latino, 18% other.  ***Education:***  0% High school or less  100% College or higher.  ***Children***  **Age:** x̄=36.8 months, SD=8.08 (range 20-46).  **Sex:** 72.7% male, 27.3% female. |
| **Ruiz-Baqués et al. 2018 [67]** | Food allergy | Quasi-experimental design: pre-test post-test design. | To evaluate the impact of an online educational program designed for parents and caregivers of children with food allergies. | ***Country:*** Spain  ***Recruitment area:*** Major cities of Spain. Most of the participants in this study were members of patient organizations. | ***Parents***  **Sex:** Female 75%, unknown 10%.  **Age: N**ot provided.  **Ethnicity**: Not provided.  **Education:** Not provided.  ***Children***  **Age:** x̄=5.46 (3.5) years.  **Sex:** 85 males, 45 females. |
