# Supplementary material for: Digital Interventions to Improve Health Literacy Among Parents of Children Aged 0 to 12 Years With a Health Condition: Systematic Review
Source: J Med Internet Res. 2021 Dec 22;23(12):e31665. doi: 10.2196/31665 (PMC8734927; doi:10.2196/31665)
Supplement: Multimedia Appendix 4 [file jmir_v23i12e31665_app4.docx]

Appendix 4 Descriptions of intervention and outcomes.

| **Author, year** | **Description of digital health intervention** | **Characteristics of intervention** | **Outcome - engagement** | **Outcome - health literacy** | **Outcome -child health** |
| --- | --- | --- | --- | --- | --- |
| **Blatz et al. 2017 [63]** | Website for mothers of preterm infants hospitalised in neonatal intensive care unit including:  1. milk diary (mothers record the time and volume of each milk expression)  2. educational materials to establish and maintain adequate milk supply,  3. developmental and kangaroo care.  LactationLogPlus was developed as an alternative to paper diaries with a readily available usable form of technology as a source for breastfeeding and breast milk expression information, and a mechanism for tracking maternal milk volumes. | ***Type*:** Website  ***Name:*** LactationLogPlus.  ***Rationale:***  To give mothers a means of active involvement in their efforts to provide breast milk for their preterm infants.  ***Length of intervention:*** >1 week.  ***Time between intervention and follow up****:* 2-3 days after discharge from the hospital or ceasing breastmilk expression. | ***Utilisation:***  Of the 13 participants, 9 indicated that they had used the breast milk expression educational information and 4 indicated they did not.  Of the 20 mothers enrolled in the study, 13 mothers logged on, 2 mothers never logged on and 5 mothers logged on to the website only when guided through the process.  The 13 users logged on to the web site from 2 to 45 times (x̄=13.3, SD=11.7). These 13 mothers entered 1 to 133 breast milk expression sessions (x̄=33.2, SD=35.4)  ***Satisfaction:*** Not measured. | ***Health knowledge:*** Not measured.  ***Health behaviour:***  Milk diary helped to pump milk  Of the 13 participants, 2 felt the milk log web site helped a great deal, 5 felt that web site log helped somewhat, 6 felt the web site log did not help them to pump breast milk.  Milk diary helped to maintain milk supply. Of the 13 the participants, 2 felt the milk log web site helped them to pump breast milk a great deal, 3 felt that web site log helped somewhat and 8 felt the web site log did not help them maintain milk supply. | ***Premature birth***  The study did not examine changes in infant breast feeding or how much breastmilk the preterm infant received. |
| **Fiks et al. 2016 [64]** | The MyAsthma provides educational material; enables sharing of families’ treatment concerns, goals, asthma symptoms, medication adherence, and side effects with the primary care clinical team.  Families can track asthma control over time using the website and clinicians through the electronic health records. This provides information and decision support to both families and clinicians regarding asthma control. | ***Type:*** Patient portal* on website.  ***Name:*** MyAsthma.  ***Rationale:***  Patient portals have the potential to facilitate the management of chronic diseases, specifically asthma.  ***Length of intervention:*** Not provided.  ***Time between intervention and follow up:***  30 days + 14 days.  * Patient portals are web-based health care applications that enable patients to interact with their health care providers on the internet. | ***Utilisation:***  Out of 9133 eligible patients, 237 (2.59%) completed the portal asthma control survey at least once (range 0.6%-13.6%). A total of 156 (65.8 % of portal users, 1.71% of eligible parents) completed the portal survey more than once (range 0.0% to 13.6%).  ***Satisfaction:*** Not measured. | ***Health knowledge:*** Not measured.  ***Health behaviour:*** Portal adopters were most likely to be taking a preventor medication at baseline, have private insurance, lower child age and greater asthma severity. Of the 156 sustained users (completion of at least two portal surveys), 76 children had uncontrolled asthma. Of the 76 children with uncontrolled asthma after the first survey, 20 (26%) had a medication change or refill within 30 days of survey completion, and 21 (28%) had an asthma-related primary care visit within 30 days. These numbers represent a significant increase in medication changes or refills and asthma-related visits when compared with the same period the year prior for each child (14% increase in medication changes [95% CI, 2%, 27%] and 16% increase in visits [95% CI, 3%, 28%]). | ***Asthma control***  The study did not examine changes in asthma control. |
| **Kobak et al. 2011 [65]** | A web-based tutorial containing three modules: an overview of autism spectrum disorder (ASD), describing the characteristics of ASD and how they impact communication and behaviour; teaching strategies including reinforcement, modelling, and prompting; and strategies for improving communication focusing on teaching communication skills to improve a child’s ability to request desired objects or activities. | **Type**: Website.  **Name**: Web-based version of Enhancing Interactions (no specific name).  ***Rationale:*** Not provided.  ***Length of intervention:*** Not provided.  ***Time between intervention and follow up:*** Not provided. | ***Utilisation:***  23 parents completed the web-based tutorials.  ***Satisfaction:***  The score on the System Usability Scale that measures satisfaction was x̄=85, SD=17, which corresponds to  a score of excellent, 52% of parents had a mean score above 90 (the highest rating possible), and 44% rated it good or excellent. No parent rated it lower than okay (scale midpoint). | ***Health knowledge:***  Parent’s knowledge of the concepts presented in the tutorial improved from pre-test to post test, increase in the number of correct items, from x̄ =12.6 to 20.4, t (23)=10.72, p<0.001, 79% scored ≥80% after taking the tutorial, compared to 8% prior to taking the tutorial.  ***Health behaviour:***  Not measured. | ***ASD***  The study did not examine changes in children’s behaviour. |
| **McGarry et al. 2020 [66]** | Participating families received 6 sessions of online training in PRT via Qualtrics based online program. The program focused specifically on promoting early language skills. The training included 5 core lessons covering key pivotal response treatment strategies and 1 review lesson. After completing each lesson, parents were asked to submit a 5-min video capturing their use of PRT strategies with their child. The submission of each video was a prerequisite for being sent a digital link for the next lesson. | **Type**: Qualtrics on website.  **Name**: Pivotal Response Treatment (PRT) an online parent training program for toddlers with autism spectrum disorder.  **Rationale:** Not provided.  ***Length of intervention:*** Mean duration of course participation was 67.27 days  (SD=43.00).  ***Time between intervention and follow up:*** Follow ups were made after each course session. | ***Utilisation:***  51 families consented to participation, 30 (58.8%) enrolled and 11 (37%) completed the program.  ***Satisfaction:***  On a 0-5 scale (0=Strongly Disagree, 5=Strongly Agree), all families reported that the course was clearly written and well organized (x̄ =4.54, SD=0.52). All families indicated that they would recommend the course to a friend (x̄ = 4.54, SD=0.52). Families reported that the course provided them with a clear understanding of PRT (x̄ =4.27, SD=0.47). Most parents reported their child’s social engagement improved (x̄ =3.91, SD=1.04). For the statement “My child’s social engagement improved over the course of intervention,” eight parents agreed, two parents strongly agreed, and one parent disagreed. Parents generally reported an improvement in their child’s language skills (x̄ =3.7, SD=1.49). | ***Health knowledge:*** Not measured.  ***Health behaviour:***  Parent’s treatment fidelity improved from baseline (x̄ =65.34%, SD=18.04) to week 5 (x̄ = 90.13%, SD=7.20); t (10) =−6.16, p<0.001. At baseline, 1 parent met fidelity of implementation (≥80% fidelity score). By the end of the program, 10 parents met fidelity of implementation, while 1 parent approached fidelity (75% fidelity score). | ***ASD***  **Child’s behaviour change** Rate of child vocalizations per minute increased from baseline (x̄ =3.84, SD=2.91) to week 5 (x̄ =5.80, SD=1.55), p=0.05. Child eye contact also increased from baseline (x̄ =7.30, SD=10.33) to week 5 (x̄ =18.68, SD=16.63), p=0.03. Child positive affect increased from baseline (x̄ =0.58, SD=0.53) to week 5 (x̄ =2.59, SD=1.58), p<0.001. |
| **Ruiz-Baqués et al. 2018 [67]** | An online educational platform with 3 units comprising educational videos including  1.general knowledge about food allergy and its symptoms and management,  2. allergen avoidance, the most common allergenic foods, labelling, cooking, shopping, and relationships with health care and  3. practical skills for proper use of an epinephrine autoinjector and mothers with children with food allergy discussed their personal experiences of various topics.  Online discussion forums moderated by physicians and expert patients within the platform were available. | ***Type:*** Online platform.  ***Name:*** Not provided.  ***Rationale:*** Digital tool can impart theoretical knowledge and practical information that can make everyday life easier.  ***Length of intervention:*** 5 hours over 2 weeks.  ***Time between intervention and follow up:*** Follow ups were made at the end of the course. | ***Utilisation:***  277 parents pre-registered, of these 207 parents enrolled in the program (74.8% engagement). Of the 207 participants who started the program, 139 (67.1%) visited the online forums: 106 (76.2%) visited the forums up to 25 times, and 33 (23.8%) more than 26 times during the 2 weeks of the program. Of the 207 participants, 130 (62.8%) completed the educational program and filled in the final post course questionnaire.  ***Satisfaction:***  Overall satisfaction with the educational program received an average rating of 8.78; in particular, 83.1% of participants gave the course a score of 8 to 10 points, and 44.6% gave it 10 points. | ***Health knowledge:***  Evaluation of knowledge acquisition pre-program and post-program found a significant improvement in 15 out of 30 questionnaire items (50%) whereof a highly significant improvement (P<.001) in 8 items (26.7%). Improvement was more frequent in the general and clinical aspects domain (7 items out of 12, 58.3%) than in the daily life domain (8 items out of 18, 44.4%).  ***Health behaviour:*** Not measured. | ***Food allergy***  The study did not examine changes in children’s symptoms or quality of life. |
